# Comparative Quantitative Aortographic Assessment of Regurgitation in Patients Treated With VitaFlow Transcatheter Heart Valve vs. Other Self-Expanding Systems

**DOI:** 10.3389/fcvm.2021.747174

**Published:** 2022-01-25

**Authors:** Rutao Wang, Hideyuki Kawashima, Chao Gao, Fangjun Mou, Ping Li, Junjie Zhang, Jian Yang, Jianfang Luo, Darren Mylotte, William Wijns, Yoshinobu Onuma, Osama Soliman, Ling Tao, Patrick W. Serruys

**Affiliations:** ^1^Department of Cardiology, Xijing Hospital, Xi'an, China; ^2^Department of Cardiology, CORRIB Research Center for Advanced Imaging and Core Laboratory, National University of Ireland, NUIG, Galway, Ireland; ^3^Department of Cardiology, Radboud University Medical Center, Nijmegen, Netherlands; ^4^Heart Center, Department of Clinical and Experimental Cardiology, Amsterdam Cardiovascular Sciences, Amsterdam UMC, University of Amsterdam, Amsterdam, Netherlands; ^5^Department of Cardiology, Yulin First People's Hospital, The Sixth Affiliated Hospital of Guangxi Medical University, Yulin, China; ^6^Department of Cardiology, Nanjing First Hospital, Nanjing Medical University, Nanjing, China; ^7^Department of Cardiovascular Surgery, Xijing Hospital, Xi'an, China; ^8^Department of Cardiology, Guangdong Cardiovascular Institute, Guangdong Provincial People's Hospital, Guangdong Academy of Medical Sciences, Guangzhou, China; ^9^The Lambe Institute for Translational Medicine and Curam, National University of Ireland Galway (NUIG), Galway, Ireland; ^10^Department of Cardiology, Imperial College London, London, United Kingdom

**Keywords:** aortic stenosis, self-expanding valve, paravalvular regurgitation, transcatheter aortic valve replacement, transcatheter heart valve, VitaFlow

## Abstract

**Objectives:**

To compare the quantitative angiographic aortic regurgitation (AR) of six self-expanding valves after transcatheter aortic valve replacement (TAVR).

**Background:**

Quantitative videodensitometric aortography (LVOT-AR) is an accurate and reproducible tool for assessment of AR following TAVR.

**Methods:**

This is a retrospective central core-lab analysis of 1,257 consecutive cine aortograms performed post-TAVR. The study included 107 final aortograms of consecutive patients who underwent TAVR with first-generation VitaFlow in four Chinese centers and 1,150 aortograms with five other transcatheter aortic valves (Evolut Pro, Evolut R, CoreValve, Venus A-Valve, and Acurate Neo). LVOT-AR analyses of these five valves were retrieved from a previously published pooled database.

**Results:**

Among 172 aortograms of patients treated with VitaFlow, 107 final aortograms (62.2%) were analyzable by LVOT-AR. In this first in man eight cases necessitated a procedural valve in valve due to inappropriate TAVR positioning and severe aortic paravalvular regurgitation. In the VitaFlow group, the mean LVOT-AR of the intermediate aortograms was 7.3 ± 7.8% and the incidence of LVOT-AR >17% was 8.6%. The mean LVOT-AR of the final aortogram was 6.1 ± 6.4% in the VitaFlow group, followed by Evolut Pro (7.3 ± 6.5%), Evolut R (7.9 ± 7.4%), Venus A-valve (8.9 ± 10.0%), Acurate Neo (9.6 ± 9.2%), and lastly CoreValve (13.7 ± 10.7%) (analysis of variance *p* < 0.001). Post hoc 2-by-2 testing showed that CoreValve had significantly higher LVOT-AR compared with each of the other five THVs. No statistical difference in LVOT-AR was observed between VitaFlow, Evolut Pro, Evolut R, Acurate Neo, and Venus A-valves. The VitaFlow system had the lowest proportion of patients with LVOT-AR >17% (4.7%) (AR after the final aortograms), followed by Evolut Pro (5.3%), Evolut R (8.8%), Acurate Neo (11.3%), Venus A-valve (14.2%), and CoreValve (30.1%) (chi-square *p* < 0.001).

**Conclusion:**

Compared to other commercially available self-expanding valves, VitaFlow seems to have a low degree of AR and a low proportion of patients with ≥moderate/severe AR as assessed by quantitative videodensitometric angiography. Once the learning phase is completed, comparisons of AR between different transcatheter heart valves should be attempted in a prospective randomized trial.

## Introduction

Moderate or severe aortic regurgitation (AR) post transcatheter aortic valve replacement (TAVR) is associated with increased long-term mortality (1, 2), therefore accurate procedural assessment of AR is critical for long term results of TAVR. Quantitative videodensitometric assessment of paravalvular leak (PVL) has been extensively vetted and validated *in-vitro* (3, 4), *in-vivo* (5), and in the clinical setting, such as after TAVR (6–18), and quantitative videodensitometric aortography is an objective, accurate, and reproducible tool for assessment of AR following TAVR that has been advocated, among other techniques, in the VARC three consensus as a reliable modality of AR assessment (19).

Aortic regurgitation severity depends on the interaction between anatomical characteristics of the native aortic valve (bicuspid leaflet, elliptical annulus, calcified cups..., etc.), on the transcatheter heart valve (THV) platform and on the implantation technique. Modifications in the design of THVs, such as radial force, sealing skirt, frame composition or size of struts, may, among others, influence the THV's sealing capacity. A wide variation in AR severity among different THVs has been reported (2–30% AR in the left ventricular outflow tract [LVOT-AR] >17%), with lower degree of LVOT-AR in THVs that feature an anti-leak skirt (10).

In the current study, first data on quantitative videodensitometric assessment of AR in TAVR patients with the VitaFlow system (MicroPort, Shanghai, China) are presented, and compared to five other self-expanding THVs.

## Methods

This is a retrospective, multi-center analysis of consecutive aortograms in 172 consecutive TAVR patients treated with the VitaFlow THV at four Chinese centers. The study complied with the Declaration of Helsinki and Good Clinical Practice guidelines and was approved by each center's ethics committees.

Videodensitometry analysis of AR was performed by two physicians of the Xijing hospital (RW and CG) and remotely supervised by an independent core laboratory (HK, MA, YO, PWS, OS at the CORRIB Corelab, NUIG, in Galway) using the CAAS A-valve 2.0.2 software (Pie Medical Imaging BV, Maastricht, the Netherlands), not financially subsidized by industry. The quantitative assessment of the AR from the aorta into the LVOT is reported as the LVOT-AR parameter. The results are expressed in percentages and quantify the fraction of AR defined as the ratio between the area under the time-density curves assessed by videodensitometry in the LVOT (region of interest, ROI) and in the aortic root (reference area) during a conventional aortography (Supplementary Figure). Technical details of videodensitometry analysis and validation in silico, *in vivo*, in animal models as well as in clinical correlations with magnetic resonance imaging, transthoracic and transesophageal echocardiography have been extensively reported in the literature (4, 12, 16, 18, 20). Post-implantation balloon dilatation improvement in AR has also been quantitatively documented in a prior study (15). Importantly, videodensitometry-derived AR has proven to be a predictor of long-term prognosis after TAVR, with a >17% threshold of AR identifying those at risk of long-term mortality (15, 18). The results of quantitative AR analyses of Evolut Pro, Evolut R, CoreValve (Medtronic, Dublin, Ireland), Venus A-valve (Venus Medtech Inc., Hangzhou, China), and Acurate Neo (Boston Scientific, Massachusetts, USA) were retrieved from a published pooled database generated by the same core laboratory (10, 12).

The first-generation VitaFlow system consists of a self-expanding nitinol frame with robust radial strength, a tri-leaflet bovine pericardial valve, and a delivery caheter that does not afford the option for valve repositioning. The double-layer (inner and outer) polyethylene terephthalate (PET) skirt at the LVOT is designed to reduce post TAVR PVL. We aimed to compare, in the six SEVs, the quantitative AR in the left ventricular outflow tract (LVOT-AR). Comparison among the THVs was performed with regard to the mean LVOT-AR (continuous variable assessment of LVOT-AR) and with regard to the proportion of patients (categorical variable) with that particular THV presenting with moderate or severe regurgitation, with a pre-determined threshold criterion of LVOTAR >17%, previously identified as moderate or severe regurgitation with respect to echocardiography (10, 17).

### Statistical Analysis

Continuous variables were reported as mean ± standard deviations. Comparison of LVOT-AR was performed using 1-way analysis of variance and 2-by-2 comparisons using the *post-hoc* Bonferroni test. Continuous variable regurgitation was stratified into categorical variables according to the following pre-determined threshold criteria: (1) none or trace (LVOT-AR <6%); (2) mild (6% to ≤ 17%); and (3) moderate or severe (>17%) (10). The proportion of patients with moderate or severe PVL (LVOT-AR >17%) was compared using a chi-square test. A 2-sided *p* value of <0.05 was considered indicative of statistical significance. Statistical analyses were performed with SPSS version 25.0 (IBM, Armonk, New York).

## Results

**Figure 1:**
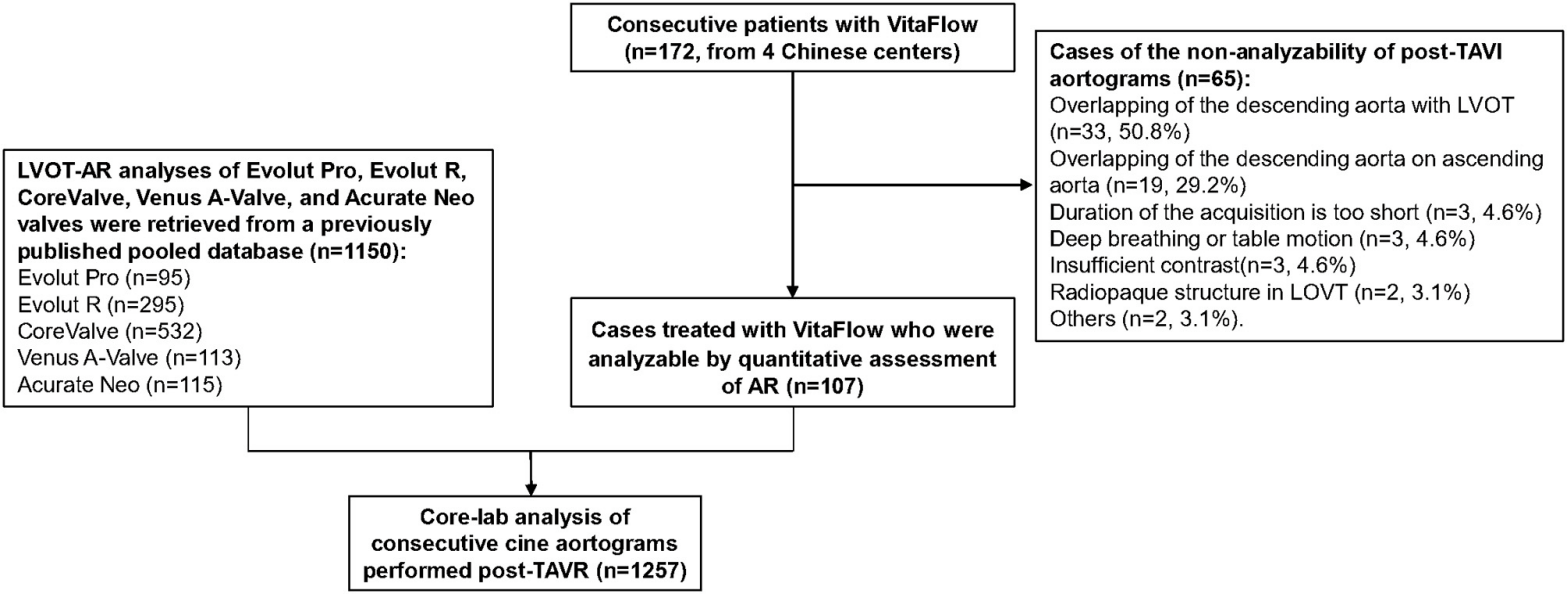
The study flow chart.

Out of 172 aortograms of patients treated with VitaFlow THV, 107 final aortograms (62.2%) were analyzable by quantitative assessment of AR. The study flow chart is shown in Figure 1. The common causes of the non-analyzability of post-TAVI aortograms are also listed in Figure 1. Out of 65 non-analyzable cases, the reasons for non-analyzable were as follows: overlapping of the descending aorta with LVOT (*n* = 33, 50.8%) overlapping of the descending aorta on ascending aorta (*n* = 19, 29.2%), duration of the acquisition is too short (*n* = 3, 4.6%), deep breathing or table motion (*n* = 3, 4.6%), insufficient contrast (*n* = 3, 4.6%), radiopaque structure in LOVT (*n* = 2, 3.1%), others (*n* = 2, 3.1%).

In this first in man experience 8 cases nessessitate a valve in valve treatment due to inappropriate positioning of the valve resulting in severe aortic paravalvular regurgitation. Among the eight valve in valve cases, only six were analyzable by videodensitometric assessment (Table 1). The two unanalyzable cases (descending aorta overlapped the LOVT) had severe regurgitation after the first valve implantation by Sellers assessment (Grade 3).

**Table 1:** Post-TAVR AR by videodensitometric assessment in valve in valve cases.

**Post-TAVR AR after first valve**	**Post-TAVR AR after second valve**
0.13	0.01
0.29	0.02
0.23	0.03
0.28	0.02
0.35	0.00
0.15	0.07
Unanalyzable (Grade 3 by Sellers assessment)	0.03
Unanalyzable (Grade 3 by Sellers assessment)	0.09

The mean LVOT-AR of the intermediate aortograms prior to the valve in valve treatment in 6 cases was 23.8 ± 8.5%, after the second valve implantation, the mean LVOT-AR of the final aortogram in these six cases was 2.5 ± 2.4%.

**Figure 2:**
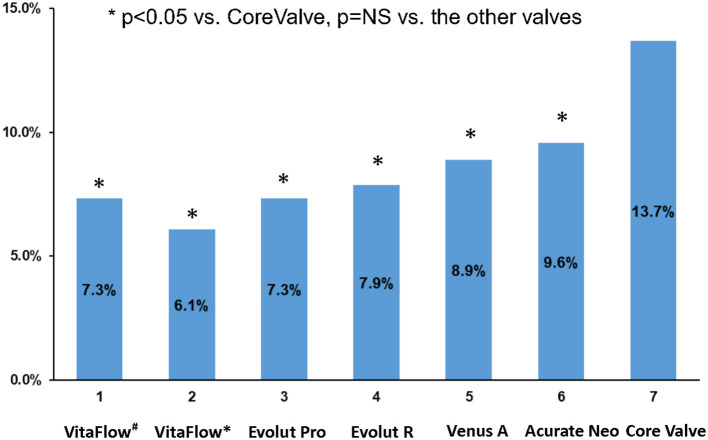
Comparison of the mean LVOT-AR after TAVR among the six THVs. VitaFlow^#^: the mean LVOT-AR of the intermediate aortograms. VitaFlow*: the mean LVOT-AR of the final aortogram (after the second valve implantation). LVOT-AR, quantitative aortic regurgitation in the left ventricular outflow tract; TAVR, transcatheter aortic valve replacement; THV, transcatheter heart valve.

The mean LVOT-AR of the intermediate aortograms was 7.3 ± 7.8% (*n* = 107) for VitaFlow. The mean LVOT-AR of the final aortogram was 6.1 ± 6.4% (*n* = 105) in the VitaFlow group followed by Evolut Pro (7.3 ± 6.5%, *n* = 95), Evolut R (7.9 ± 7.4%, *n* = 295), Venus A-valve (8.9 ± 10.0%, *n* = 113), Acurate Neo (9.6 ± 9.2%, *n* = 115), and lastly CoreValve (13.7 ± 10.7%, *n* = 532) (analysis of variance *p* <0.05) (Figure 2). Post hoc 2-by-2 testing showed that CoreValve had significantly higher LVOT-AR compared with each of other five THVs. Apart from the CoreValve, no difference in LVOT-AR was observed between VitaFlow, Evolut Pro, Evolut R, Venus A-valve, and Acurate Neo (Figure 2).

**Figure 3:**
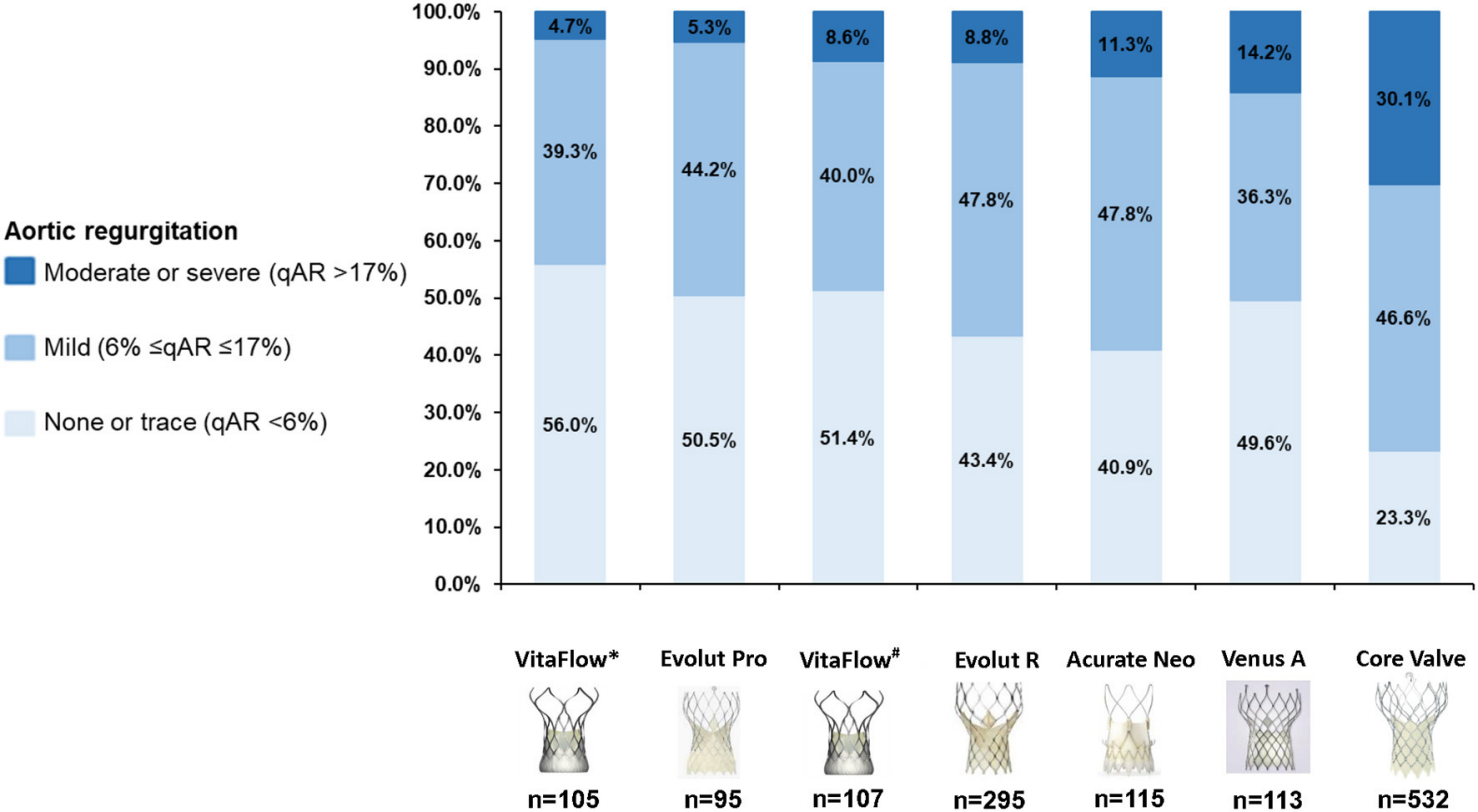
Cumulative percentage of different degrees of post-TAVR AR by videodensitometric assessment. VitaFlow*: the analysis of LVOT-AR using the final aortograms (after the second valve implantation), VitaFlow^#^: the analysis of LVOT-AR using the intermediate aortograms. AR, aortic valve regurgitation; TAVR, transcatheter aortic valve replacement.

The proportion of patients with LVOT-AR >17% (AR after the intermediate aortograms) was 8.6% in the VitaFlow group. If the final aortograms was analyzed VitaFlow THV had the lowest proportion of patients with LVOT-AR >17% (4.7%) followed by Evolut Pro (5.3%), Evolut R (8.8%), Acurate Neo (11.3%), Venus A-valve (14.2%), and CoreValve (30.1%) (chi-square *p* < 0.001) (Figure 3).

**Figure 4:**
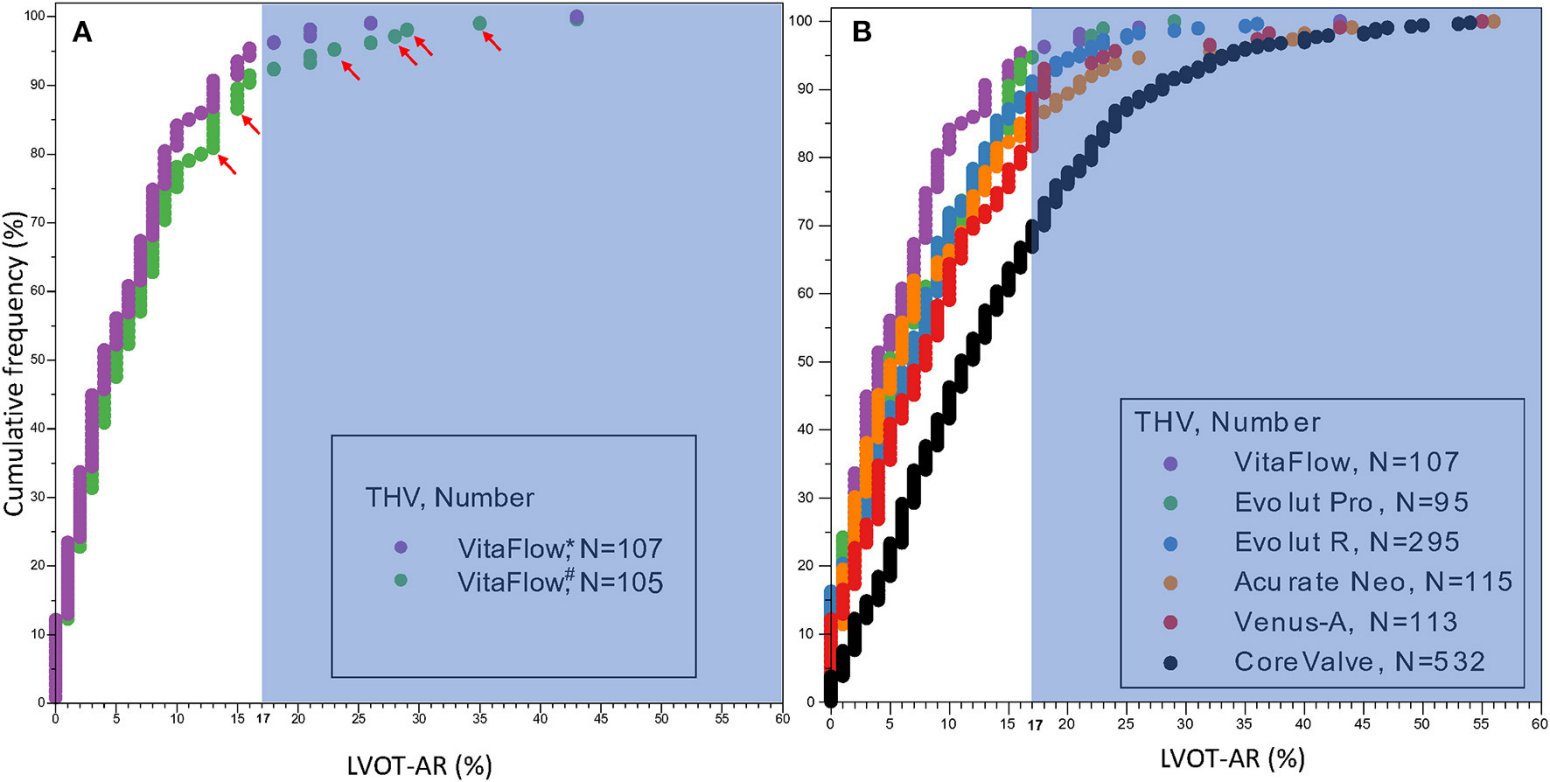
Cumulative frequency curves of LVOT-AR after TAVR for VitaFlow **(A)** and the six THVs **(B)**. The shaded background shows the area above 17% of AR, indicating moderate or severe regurgitation. LVOT-AR, quantitative aortic regurgitation in the left ventricular outflow tract; TAVR, transcatheter aortic valve replacement; THV, transcatheter heart valve. VitaFlow*: the analysis of LVOT-AR using the final aortogram (after the second valve implantation), VitaFlow^#^: the analysis of LVOT-AR using the intermediate aortograms. The red arrows show the LVOT-AR in the six valve-in-valve cases using the intermediate aortograms.

The cumulative frequency curves of LVOT-AR after TAVR for the six THVs are shown in Figure 4.

## Discussion

This is the first in man study comparing by quantitative aortography the degree of acute regurgitation following TAVR with VitaFlow in comparison with multiple cohorts of consecutive “real world” patients treated with other SEVs. The main findings of the present study are as follows:

(1) The VitaFlow THV, in comparison with other self-expanding percutaneous valve analyzed in our database, showed numerically low mean degree of LVOT-AR. The first-generation CoreValve had the highest mean LVOT-AR among the evaluated valves.
(2) The VitaFlow THV had a low proportion of patients with LVOT-AR >17% compared with other SEVs analyzed in our database.
(3) The four contributing centers were in their learning phase, which could explain the high incidence of procedural valve in valve implantation in order to correct a severe aortic paravalvular regurgitation due to malpositioned implantation in the initial attempt.

Paravalvular regurgitation, even in those with mild PVL, following TAVR is associated with mortality (2, 18, 21, 22). Accurate, objective, reproducible, and quantitative assessment of AR following TAVR is of considerable importance in assessing online the immediate hemodynamic performance of a TAVR procedure considering the detrimental long-term prognosis of any level of regurgitation.

The Seller's visual grading (23) of aortic root angiography is the first screening tool used in most laboratories for detection of post-implantation AR and guidance of timely corrective measures (e.g., post-dilation, valve-in-valve and, most recently, retrieval and reposition of the valve). However, the Seller's classification of AR is a subjective categorical evaluation and is thereby poorly reproducible. Although, evaluation of residual AR by transthoracic echocardiography (TTE) immediately post procedure has been recommended in guidelines, the acquisition of TTE in the cath-lab in a prone position is challenging. TEE is even more challenging since it requires a general anesthesia that has largely been abandoned in the worldwide practice of TAVR.

Furthermore, echocardiographic assessment of regurgitation is semiquantitative, operator dependent, and not reproducible, even when performed by core laboratories (24). Previous studies have demonstrated that TAVR performed exclusively under angiographic guidance with back-up TTE is feasible and associated with reasonably good outcomes, similar to those of angiography and transesophageal echocardiography-guided procedures (25). Based on previous validations (4, 10, 12), quantitative aortographic assessment of AR is an objective, accurate, and reproducible tool for adjudication of AR following TAVR and the Academic Research Consortium on percutaneous valve replacement (VARC 3) has recently listed the videodensitometric technique as a reliable modality of assessment of aortic regurgitation (19). Moreover, quantitative aortography has the potential for periprocedural TAVR guidance by facilitating timely decision-making to avert AR using balloon post-dilatation, retrieval-reposition, or valve in valve implantation. In our present study, there are eight cases (six of them could be analyzed by videodensitometric assessment) necessitating a valve in valve treatment due to inappropriate positioning of the valve resulting in severere regurgitation. After valve-in-valve implantation, the mean LVOT-AR of the final aortogram in these six cases was much lower than the mean LVOT-AR of the intermediate aortogram prior to the valve in valve treatment (2.5 ± 2.4 vs. 23.8 ± 8.5%).

**Figure 5:**
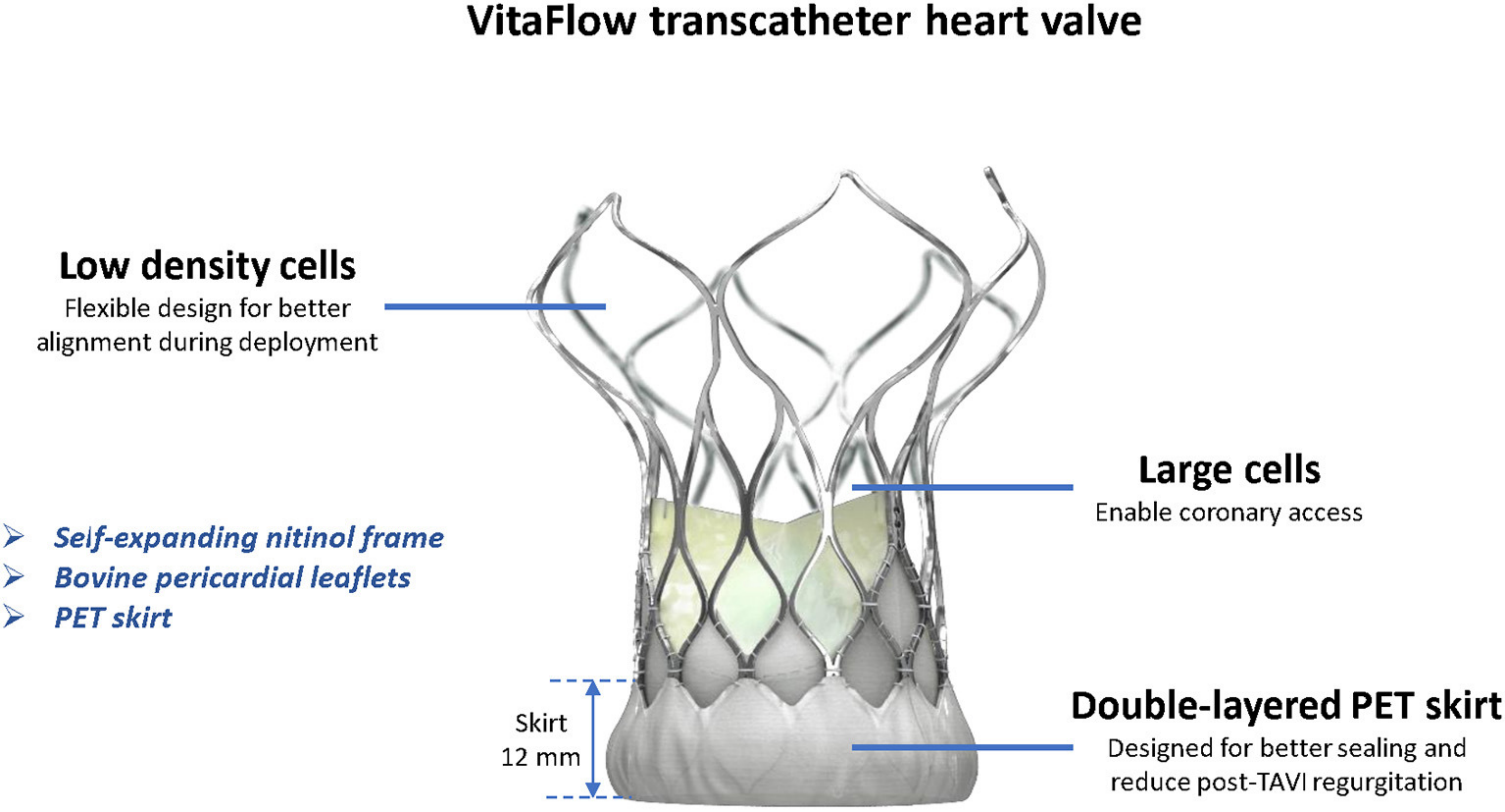
The VitaFlow transcatheter aortic valve system.

The first-generation VitaFlow THV is a novel Chinese transcatheter valve system with a unique combination of characteristics such as a self-expanding nitinol frame with high-radial force, a double-layered PET skirt, and features the only commercially available motorized delivery system worldwide (Figure 5; Table 2).

**Table 2:** Design features of the six THVs and their performance on post TAVR acute AR by videodensitometry analysis.

**TAVI device**	**Manufacture**	**Sheath size in F**	**Size in mm**	**Material**	**External pericardial wrap**	**Other features and limitations**	**The mean LVOT-AR**	**The proportion of patients with LVOT-AR >17%**
VitaFlow	MicroPort, Shanghai, China	16/18	21, 24, 27, 30	Bovine pericardium tissue valve. SE nitinol frame.	Yes	Not recapturable, not repositionable. Design with high radial force.	7.3 ± 7.8% (n=107) 6.1 ± 6.4% (*n* = 105)	8.6% (*n* = 107) 4.7% (*n* = 105)
Evolut Pro	Medtronic Inc.,Dublin, Ireland	16	23, 26, 29	Porcine pericardium tissue valve. SE nitinol frame.	Yes	Recapturable, retrievable, repositionable. Does not require rapid pacing for deployment. 10% smaller in height than CoreValve.	7.3 ± 6.5% (*n* = 95)	5.3% (*n* = 95)
Evolut R	Medtronic Inc.,Dublin, Ireland	14/16	23, 26, 29, 34	Porcine pericardium tissue valve. SE nitinol frame.	No	Recapturable, retrievable, repositionable. Does not require rapid pacing for deployment. 10% smaller in height than CoreValve.	7.9 ± 7.4% (*n* = 295)	8.8% (*n* = 295)
Venus-A	Venus Medtech Inc., Hangzhou, China	19	23, 26, 29, 32	Porcine native aortic leaflets. SE nitinol frame.	No	Not retrievable, not repositionable. Designed with increased radial force at the initial 20 mm of the stent inflow segment. Has 3 positioning marker.	8.9 ± 10.0% (*n* = 113)	11.3% (*n* = 113)
Acurate Neo	Boston Scientific, Massachusetts, USA	18	Small (21–23), Medium (23–25), Large (25–27)	Porcine native aortic leaflets. SE nitinol frame.	No	Not retrievable, partially repositionable. Does not require fast pacing. Safe two-step, top-down deployment with stable hemodynamics.	9.6 ± 9.2% (*n* = 115)	14.2% (*n* = 115)
CoreValve	Medtronic Inc.,Dublin, Ireland	18/20	26, 29, 31	Porcine pericardium tissue valve. SE nitinol frame.	No	Not recapturable, not retrievable. Repositionable, does not require rapid pacing for deployment.	13.7 ± 10.7% (*n* = 532)	30.1% (*n* = 532)

It was granted China's National Medical Products Administration approval in July 2019.

In the current study, the VitaFlow THV, in comparison with the other commercially available SEVs analyzed in our database, showed a low degree of mean LVOT-AR and had a low (4.7%) occurrence of moderate or severe PVL (>17 % LVOT-AR). This is in line with the previous study reporting that only 2% of patients had moderate PVL (assessed by echocardiography) at discharge and at 30 days (26). Of note at 1-year, there was no incidence of moderate or severe PVL (26). The low rates of moderate to severe PVL could be partially explained by the double-layered anti-leak skirt of the VitaFlow THV on the valve frame. Similarly, Evolut Pro which also has an external skirt on the valve frame, showed a good performance in post TAVR PVL in our analysis. The external skirt contributes to minimizing AR by facilitating the plugging of micro-channels at the THV anchor site. To mitigate PVL after TAVR, anti-leak skirt is becoming a “must” in the development of the new generation SEV (Table 2). The present findings should be confirmed in prospective randomized comparisons of AR between different THVs.

Post-TAVR AR has been associated with mortality and adverse events (2, 18, 21, 22); it may increase the medical cost related to the subsequent potential adverse events caused by AR. Therefore, to assess AR accurately and compare the performance of AR after TAVR between different valves are crucial and may guide the device selection in clinical practice. Moreover, compared to traditional AR assessment tools such as echocardiography and cardiac magnetic resonance, VD-AR assessment was accurate, fast, and saved the cost. New trials (OVAL GUIDE Europe, OVAL GUIDE China, OVAL GUIDE Japan) are currently in preparation to evaluate the feasibility and efficacy of the VD guidance during TAVR.

Although this is the first data evaluating the quantitative videodensitometric assessment of AR in TAVR patients treated with the first-generation VitaFlow THV, our retrospective study had several limitations. Firstly, the analyzability of post-TAVI aortograms by quantitative assessment was not high due to a lack of acquisition protocol (Supplementary Material) in a retrospective study. However, in both the ASSESS-REGURGE (12) and the OVAL (6) studies, the analyzability in prospective acquisition reached more than 90%. Second, only the acute performance of AR following TAVR was reported in our retrospective analysis. The main clinical and procedural characteristics of study populations were not collected. Clinical outcomes and long-term performance of the AR have not been investigated. Moreover, the antithrombotic strategies during/after TAVR which are associated with the long-term function of valve prosthesis and may impact AR, were not available in the present study. Although the evidence for optimal antithrombotic strategies has not been fully elucidated, a recently published state-of-the-art review recommends clinicians for practicing precision medicine by integrating evidence-based knowledge and patient needs to individualize treatment strategies and keep the balance between thrombotic and hemorrhagic risks (27). Third, the aim of the current study was to compare the different quantitative degrees of regurgitation among the THVs. Thus, no information regarding calcification, presence of bicuspid valves, aortic annular size and shape, THV diameter, adequacy of sizing, technique, and depth of implantation was collected. We did not collect echocardiographic data in the present study. There was no direct comparison between quantitative videodensitometric assessment of AR and echocardiographic evaluation in our analysis. However, previous studies have demonstrated that VD-AR >17% corresponded to moderate or severe AR in comparison with trans-thoracic or trans-esophageal echocardiogram, and was associated with an increase in mortality after TAVR (13, 17). Moreover, in the VARC-3 criteria recently published (19), AR by videodensitometry is acknowledged as a valid quantitative assessment, although doppler echocardiography remains by tradition and convention the primary modality for assessing and comparing regurgitation after TAVR. Finally, the present pilot study involving four sites in their learning phase is a retrospective analysis, without randomized comparison and with a limited sample size. Prospective, multi-center, randomized, head-to-head comparisons of AR and clinical outcomes between different THVs in larger scale trials are urgently warranted in the future and indeed, is currently ongoing (ClinicalTrials.gov NCT04275726) (8).

## Conclusions

Compared to other commercially available self-expanding THVs, the VitaFlow THV seems to have a low degree of AR and a low proportion of patients with ≥moderate/severe AR as assessed by quantitative videodensitometric angiography. These results should be confirmed in prospective randomized comparisons of AR of different THV types.

## Data Availability Statement

The raw data supporting the conclusions of this article will be made available by the authors, without undue reservation.

## Ethics Statement

The studies involving human participants were reviewed and approved by Xijing Hospital. Written informed consent for participation was not required for this study in accordance with the national legislation and the institutional requirements.

## Author Contributions

PS and LT had full access to all of the data in the study and takes responsibility for the integrity of the data and the accuracy of the data analysis and including and especially any adverse effects. RW, CG, and HK contributed to the data collection, analysis and interpretation, and the writing of the manuscript. FM, JZ, PL, JY, JL, and LT contributed to the data collection and analysis and interpretation. DM, WW, YO, and OS contributed substantially to the data analysis and interpretation and the critical revision of the manuscript. All authors contributed to the article and approved the submitted version.

## Funding

This work RW, CG, and WW were supported by Science Foundation Ireland Research Professorship Award (15/RP/2765).

## Conflict of Interest

PS reports consultant fees from Merillife, Novartis, Philips/Volcano, SMT, Sinomedical Sciences Technology, Xeltis outside the submitted work. DM is a proctor and consultant for Microport, Boston Scientific, and Medtronic. WW is co-founder of Argonauts, medical advisor Rede Optimus Research and reports honoraria from MicroPort. The remaining authors declare that the research was conducted in the absence of any commercial or financial relationships that could be construed as a potential conflict of interest.

## Publisher's Note

All claims expressed in this article are solely those of the authors and do not necessarily represent those of their affiliated organizations, or those of the publisher, the editors and the reviewers. Any product that may be evaluated in this article, or claim that may be made by its manufacturer, is not guaranteed or endorsed by the publisher.
