# Supplementary material for: Comparative Quantitative Aortographic Assessment of Regurgitation in Patients Treated With VitaFlow Transcatheter Heart Valve vs. Other Self-Expanding Systems
Source: Front Cardiovasc Med. 2022 Jan 25;8:747174. doi: 10.3389/fcvm.2021.747174 (PMC8821967; doi:10.3389/fcvm.2021.747174)
Supplement: Supplementary file 1 [file Data_Sheet_1.docx]

**The acquisition protocol for the final aortogram after TAVR procedure**

**Acquisition parameters:**

1. The dynamic control optimization should be turned off. In some Cath labs it relates to blocking the KV. Please use the LV angiography protocol instead of the coronary angiography protocol when the final aortography is performed.

2. The angiogram should be performed with the catheter no more than 2 cm above the –plane of the cusps of the aortic prosthesis.

3. The catheter should be at least a 5F pigtail catheter.

4. Ideally the LV guidewire should be taken out before the injection – not mandatory.

5. Injection parameters should be of 20 mL /sec and at a pressure limit of 1200 psi.

6. The volume can be left to the operators’ discretion, no lower than 8 mL, ideally from

12 to 20 mL.

7. The patient must be in complete apnea in mid-inspiration BEFORE starting the acquisition of the image.

8. Radiopaque structures should not be overlapping with the left ventricle outflow tract, nor with the aortic root.

9. The injection should start after at least 1 cardiac cycle of image acquisition.

10. Image recording should last until all contrast is washed away from the aortic root (and at least for 3 heart beats after injection.

11. The table should not move during acquisition (even before starting the injection – i.e. the position of the heart cannot change during the entire acquisition).

**Projection for acquisition:**

1. The acquisition should be performed in a way that neither the left ventricle outflow tract nor the aortic root overlap with the descending aorta. That can be performed by simple visual assessment or by pre-planning with CT planning images. See examples of acquisition in **Figures 1** and **2**.

2. There is no need for the cusps of the aortic valve to be aligned for this acquisition!

3. A practical plan would be to angulate C-arm to right anterior oblique of more than 20 degrees or left anterior oblique of more than 30 degrees.


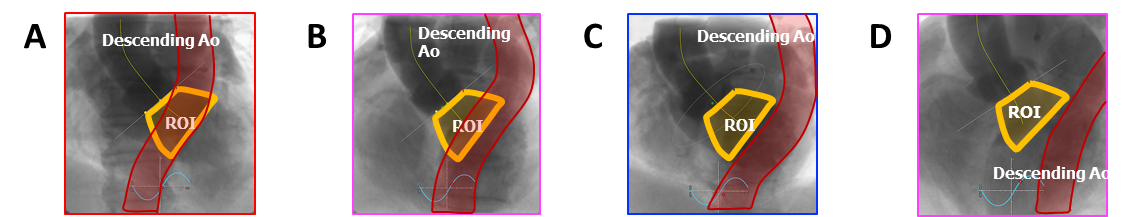


**Figure 1.** Examples of acquisition projections. In A and B there is overlapping of the descending aorta with the region of interest (ROI) – left ventricle outflow tract. In C and D examples of the proper projections to acquire, avoiding overlap of the ROI with the descending aorta.


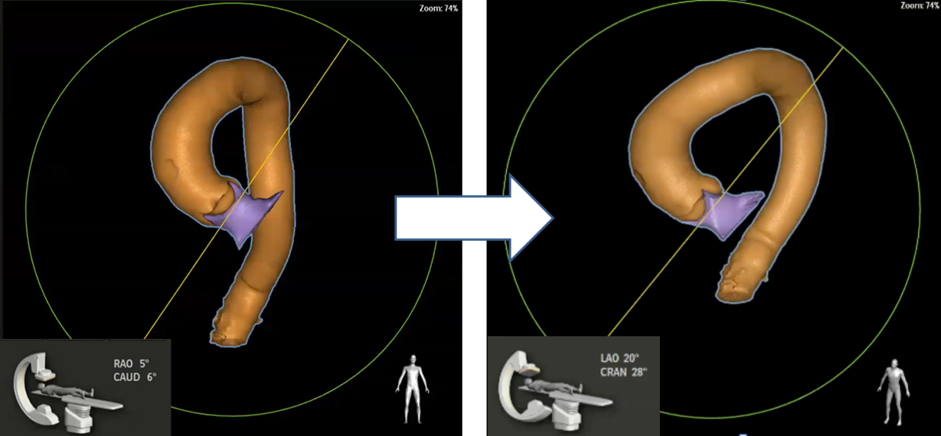


**Figure 2:** Example of CT pre-planning image. The purple segment (left ventricle outflow tract) cannot overlap with the descending aorta (left panel). Angulating the C-arm to LAO (20 degrees) is enough to acquire a proper image without overlapping.
