# Supplementary figures and images for: Comparative Quantitative Aortographic Assessment of Regurgitation in Patients Treated With VitaFlow Transcatheter Heart Valve vs. Other Self-Expanding Systems
Source: Front Cardiovasc Med. 2022 Jan 25;8:747174. doi: 10.3389/fcvm.2021.747174 (PMC8821967; doi:10.3389/fcvm.2021.747174)

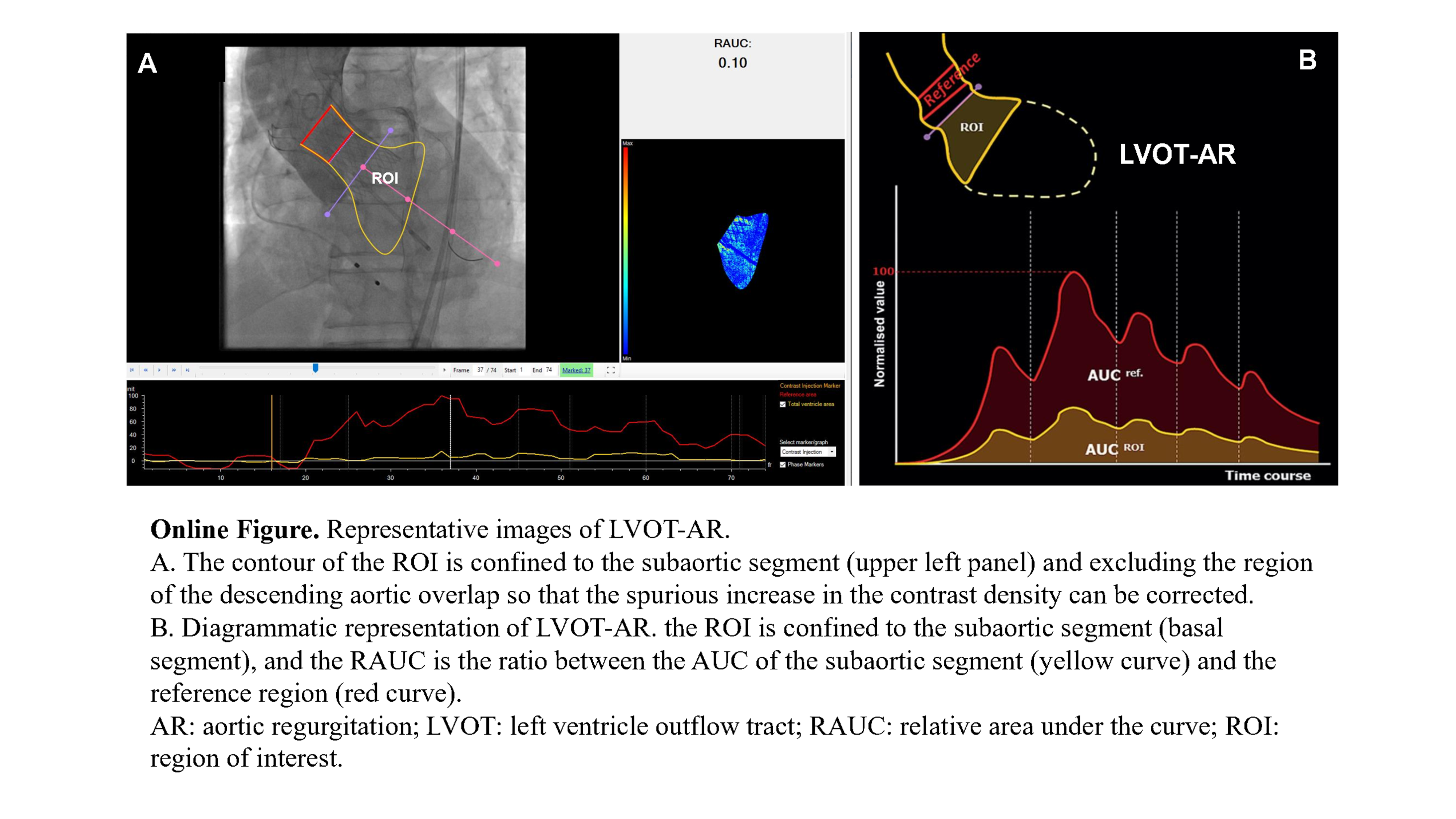

Supplement: Supplementary file 2 [file Image_1.TIFF]
